# Supplementary material for: Natural Rubber/Hexagonal Mesoporous Silica Nanocomposites as Efficient Adsorbents for the Selective Adsorption of (−)-Epigallocatechin Gallate and Caffeine from Green Tea
Source: Molecules. 2023 Aug 11;28(16):6019. doi: 10.3390/molecules28166019 (PMC10458317; doi:10.3390/molecules28166019)
Supplement: Supplementary file 1 [file molecules-28-06019-s001.zip › molecules-2499595-supplementary.pdf]

Supporting Information

Natural rubber/hexagonal mesoporous silica nanocomposites as efficient adsorbents for the selective adsorption of (−)-epigallocatechin gallate and caffeine from green tea

Kamolwan Jermjun [1], Rujeeluk Khumho [2], Mookarin Thongoiam [2], Satit Yousatit [2], Toshiyuki Yokoi [3], Chawalit Ngamcharussrivichai [2,4,5] and Sakdinun Nuntang [1,*]

[1] Industrial Chemistry Innovation Program, Faculty of Science, Maejo University, Chiang Mai 50290, Thailand; kamol.nm@hotmail.com
[2] Department of Chemical Technology, Faculty of Science, Chulalongkorn University, Bangkok 10330, Thailand; rujeeluk_fai_@hotmail.com (R.K.); mookarinth@gmail.com (M.T.); y.satit@hotmail.com (S.Y.); chawalit.ng@chula.ac.th (C.N.)
[3] Chemical Resources Laboratory, Tokyo Institute of Technology, Yokohama 226-8503, Japan; yokoi@cat.res.titech.ac.jp
[4] Center of Excellence on Petrochemical and Materials Technology (PETROMAT), Chulalongkorn University, Bangkok 10330, Thailand
[5] Center of Excellence in Catalysis for Bioenergy and Renewable Chemicals (CBRC), Faculty of Science, Chulalongkorn University, Bangkok 10330, Thailand
* Correspondence: sakdinun.nt@gmail.com

Table S1. Textural properties and water adsorption volumes of pure-silica HMS and NR/HMS nanocomposites

Sample[a]	S_{BET}[b] (m^2 g^{-1})	D_p[c] (*nm*)	V_t[d] (cm^3 g^{-1})	V_m[e] (cm^3 g^{-1})
HMS-C_{10}	781	2.49	1.04	67.20
HMS-C_{12}	692	2.89	1.10	64.97
HMS-C_{14}	677	3.46	1.40	62.91

NR/HMS-C_{10}	638	2.12	1.34	42.90
NR/HMS-C_{12}	661	2.37	1.44	44.76
NR/HMS-C_{14}	668	2.82	1.04	46.56

[a]Extracted samples

[b]BET surface area

[c]Pore diameter, calculated by BJH method

[d]Total pore volume, determined by volume adsorbed at P/P_0 = 0.99

[e]Monolayer adsorption volume at standard temperature and pressure, determined from H_2O adsorption–desorption measurement

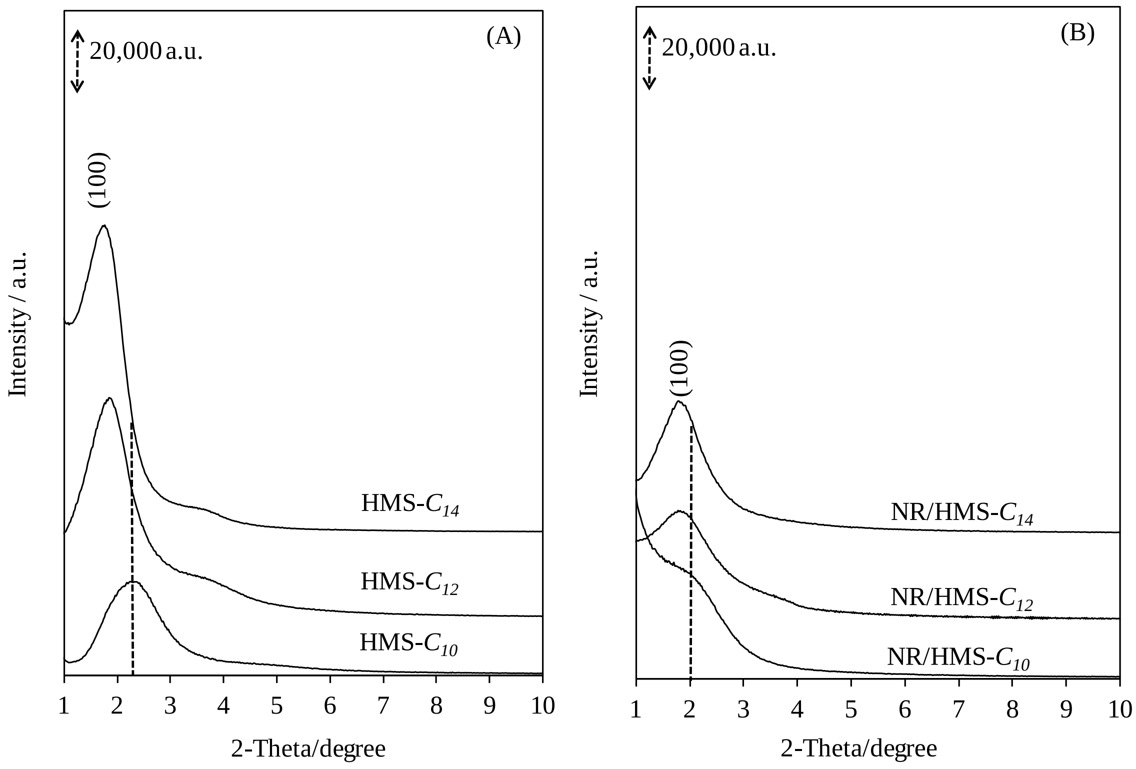

Figure S1. XRD patterns of (A) pure-silica HMS materials and (B) NR/HMS nanocomposites.

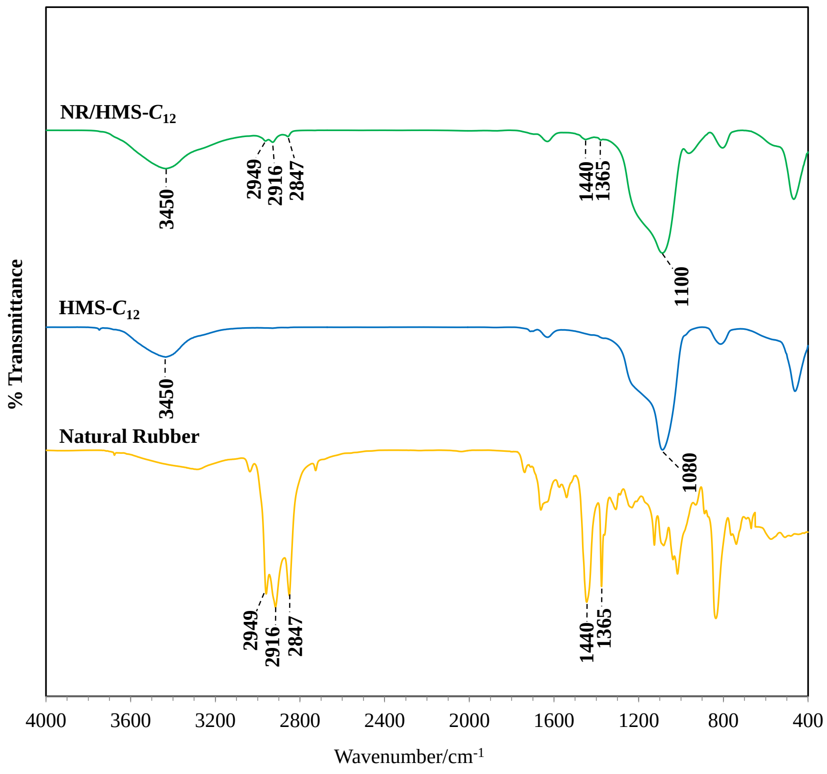

Figure S2. FTIR spectra of Natural Rubber, (b) HMS-C_{12} and (c) NR/ HMS-C_{12} composite.

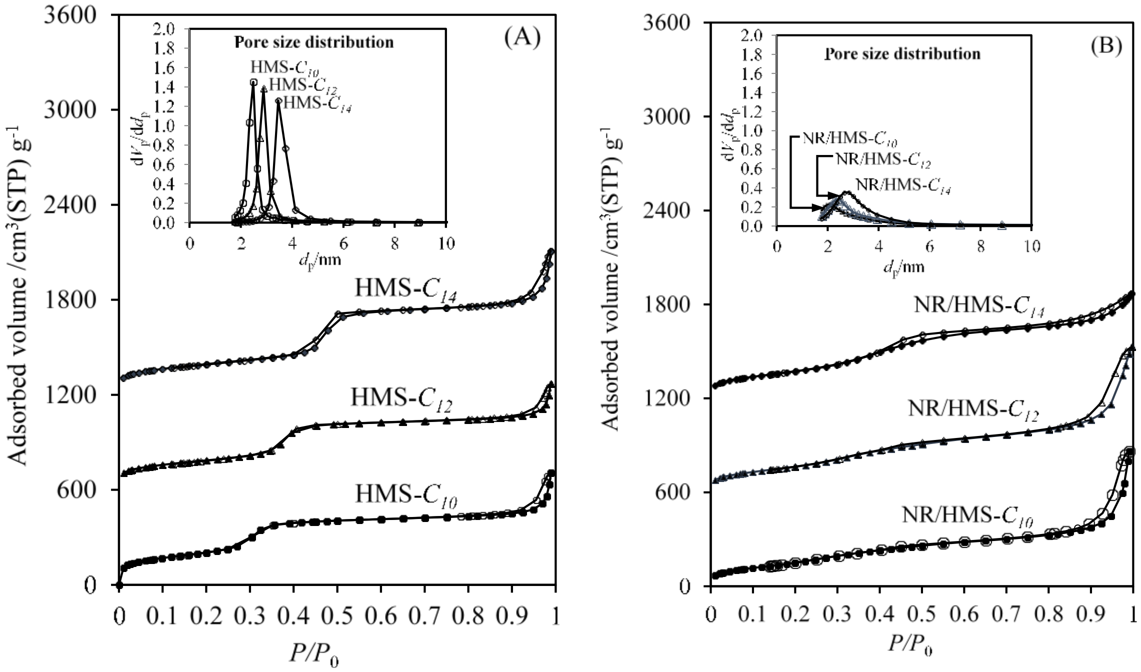

Figure S3. N_2 adsorption–desorption isotherms and BJH pore size distribution of (A) pure-silica HMS materials and (B) NR/HMS nanocomposites.

- *Adsorption study*

Equation (S1) was used to calculate the adsorption capacity (q_e).

Adsorption capacity:

$$q_e = \frac{(C_0 - C_e)V_i}{W} \qquad \text{(S1)}$$

where q_e is adsorption capacity at adsorption equilibrium (mg/$g_{dried\ adsorbent}$); C_0 and C_e are the initial and equilibrium concentrations, respectively, of EGCG or CAF in the solution (mg/L); V_i is the volume of the initial solution (mL); and W is the weight of the dried adsorbent (g).

- *Kinetic models*

Pseudo-first-order, pseudo-second-order, and intraparticle diffusion kinetic models of the experiment data were evaluated according to Eqs. (S2)–(S4) to postulate the mechanism of EGCG and CAF adsorption on the synthesized adsorbents.

Pseudo-first-order model:

$$q_t = q_e\,(1 - e^{-K_1 t}), \tag{S2}$$

pseudo-second-order model:

$$q_t = K_2 \frac{q_e^2 t}{1 + K_2 q_e t} \tag{S3}$$

intraparticle diffusion model:

$$q_t = K_p t^{0.5}, \tag{S4}$$

where q_t is the adsorption capacity at any time (mg/$g_{\text{dried adsorbent}}$); K_1 (/min) and K_2 (g/(mg min)) are the rate constants of the pseudo-first-order and pseudo-second-order models, respectively; K_p is the intraparticle diffusion rate constant (mg/g min$^{1/2}$).

- *Desorption study*

Equations (S5) and (S6) were used to calculate the desorption capacity and desorption ratio.

Desorption capacity:

$$q_d = \frac{C_d V_d}{W} \tag{S5}$$

desorption ratio:

$$D = \frac{C_d V_d}{(C_o - C_e) V_i} \times 100\% \qquad \text{(S6)}$$

where q_d is the desorption capacity after adsorption equilibrium (mg/$g_{dried\ adsorbent}$); C_d is the concentration of EGCG or CAF in the desorption solution (mg/L); V_i and V_d are the volumes of the initial and desorption solution (mL), respectively; D is the desorption ratio; and W is the weight of the dry adsorbent (g).
